# Mycoplasma Pneumoniae Induced Rash and Mucositis with Bilateral Otitis Media and Sinusitis

**DOI:** 10.7759/cureus.7449

**Published:** 2020-03-28

**Authors:** Daniel H Lofgren, Christopher Lenkeit, Jaishree Palanisamy, Jens Brown

**Affiliations:** 1 Otolaryngology, McLaren Oakland Hospital, Pontiac, USA

**Keywords:** mycoplasma pneumoniae induced rash and mucositis, mycoplasma, mycoplasma pneumoniae, otitis media, sinusitis, mirm, mucositis, rash

## Abstract

Mycoplasma pneumoniae induced rash and mucositis (MIRM) is a recently identified clinical entity, which describes a subset of extrapulmonary manifestations resulting from Mycoplasma pneumonia infection. Patients present with a wide variety of symptoms including cough, dyspnea, mucositis, conjunctivitis, with or without a variable cutaneous rash. A 24-year-old male presented to the emergency department with worsening dyspnea and new-onset oral, ocular, and genital mucosal lesions. The patient was also found to have bilateral otitis media with tympanic membrane rupture and ethmoid sinusitis upon further evaluation. The patient was originally diagnosed with atypical pneumonia leading to acute hypoxic respiratory failure and was admitted to inpatient care. Work-up revealed positive Mycoplasma pneumoniae immunoglobulin M, and the patient was subsequently diagnosed with MIRM. The patient was provided with supportive care as well as systemic antibiotics, and he fully recovered by day 12 without complication. No standardized treatment guidelines exist for MIRM, and it is universally accepted that supportive management is the mainstay of treatment, consisting of pain management, intravenous hydration, and mucosal care. Although the majority of MIRM patients are generally known to have a full recovery (81%), a variety of ocular, oral, and genital complications have been noted in the literature. Here we present a unique case of MIRM in a 24-year-old male who also had ethmoid sinusitis and bilateral otitis media with unilateral tympanic membrane perforation - two head and neck symptoms not described in previous literature.

## Introduction

*Mycoplasma pneumoniae *(MP) is an atypical respiratory pathogen that can commonly result in community-acquired pneumonia (CAP) and has an overall prevalence of 10.1% in CAP patients [1]. It has been reported that approximately 25% of patients diagnosed with MP experience extrapulmonary manifestations [2-4]. Dermatologic manifestations include urticaria, erythema multiforme (EM), Steven-Johnson syndrome (SJS), and toxic epidermal necrolysis (TEN) [2]. Recently, a new disease classification has been presented in the literature within the spectrum of EM/SJS/TEN and is associated with MP. In 2014, Canavan et al. performed the largest systematic review to date (n=202) of patients suffering from mucocutaneous lesions with positive MP testing and were the first to coin the term *Mycoplasma pneumoniae* induced rash and mucositis (MIRM) [5]. The pathophysiology of MIRM is still not fully understood, and multiple mechanisms have been proposed. The primary reported theory suggests cloning of B cells with cutaneous immune complex deposition and complement formation leading to localized inflammation and destruction. Molecular mimicry between *Mycoplasma* adhesion molecules and keratinocyte antigen has also been proposed [3-7].

Clinically, Canavan et al. noted that MIRM can present with varying degrees of mucosal involvement with or without cutaneous involvement. Patients presented with characteristic vesiculobullous and/or atypical targetoid eruptions, and, in almost every case, mucositis of the oral cavity, orbit, and/or genitalia. They also reported that MIRM correlated with a milder disease course, low rates of sequelae, and lower mortality than EM, SJS, or TEN [3,5]. Most patients suffering from MIRM are pediatric males, as seen in the emergency department for generalized and pulmonary symptoms, who then further develop cutaneous complications. It appears that these patients have 7 to 10 days of prodromal fever, malaise, and cough before mucocutaneous eruption [3,5-7]. Canavan et al. noted sparse cutaneous involvement in 47% of patients compared with severe mucositis alone (34%) and moderate cutaneous involvement alone (19%). Cutaneous involvement has been shown to present more acrally (46%) compared with both truncal (23%) and generalized locations (31%) [5,8]. The cutaneous lesions varied in appearance from vesiculobullous (77%) to targetoid (48%), papular (14%), macular (12%), and morbilliform (9%) [3-5]. In a study of 152 children with CAP, 44 (28.9%) tested positive for MP, and of these children, 10 (22.7%) developed mucocutaneous lesions [6]. The hallmark of MIRM is diffuse mucositis and involves most commonly the oral cavity (94%), with lesions consisting of erosions, ulcers, and denuded tissue. Ocular involvement, including purulent bilateral conjunctivitis, photophobia, pseudomembrane formation, ulceration, and eyelid edema, occurs in 82% of patients. Urogenital lesions noted as erosions and ulcerations of the genitals and anus are seen in 63% of patients [3-5].

MIRM is traditionally diagnosed after the eruption of mucocutaneous lesions, and initial work-up generally starts in the emergency department for pulmonary symptoms. A chest X-ray can reveal any type of atypical lung pattern including reticulonodular densities, interstitial infiltrates, and lobar consolidation [7-9]. Patients can also have elevated C-reactive protein (CRP), erythrocyte sedimentation rate (ESR), and leukocytosis with left shift [3,4,6,7]. Serologic testing for identification can include cold agglutinins, polymerase chain reaction (PCR), enzyme-linked immunoassays (ELISA), and MP immunoglobulin (Ig) M antibody levels ].

In 2014, a diagnostic criterion was proposed to identify both the classic and subtypes of MIRM. Classic MIRM includes clinical and laboratory evidence of atypical pneumonia caused by *Mycoplasma* with the following: two or more involved mucosal sites, less than 10% involved cutaneous surface area, few vesiculobullous lesions, or atypical scattered targets ± targetoid lesions. The two variants of MIRM coined were severe MIRM, which showed extensive involvement of atypical targetoid lesions or blisters, and MIRM sine rash, which showed minimal morbilliform lesions with few vesicles [3,5,10]. Interestingly, patients with MIRM sine rash had higher rates of mucosal involvement: oral (100%), ocular (92%), and urogenital (78%) [3,5].

In the next section, we present a new case of MIRM in an adult patient who presented with two new head and neck symptoms that have never been reported in the literature in either the adult or pediatric populations. The authors hope that proper evaluation of all mucosal surfaces, specifically within the head and neck, can lead to an earlier diagnosis of MIRM.

## Case presentation

A 24-year-old male, with no prior past medical history, presented to the emergency department with one week of cough and shortness of breath. Symptoms initially began with nasal congestion and left-sided otalgia after working in a dirty warehouse. Over the next 24 hours, the patient noted worsening lethargy and acute-onset dyspnea. Within the coming days, he developed diffuse oral pain with associated odynophagia to both liquids and solids. After further questioning, the patient noted unilateral bloody otorrhea after blowing his nose earlier in the day.

**Figure 1:**
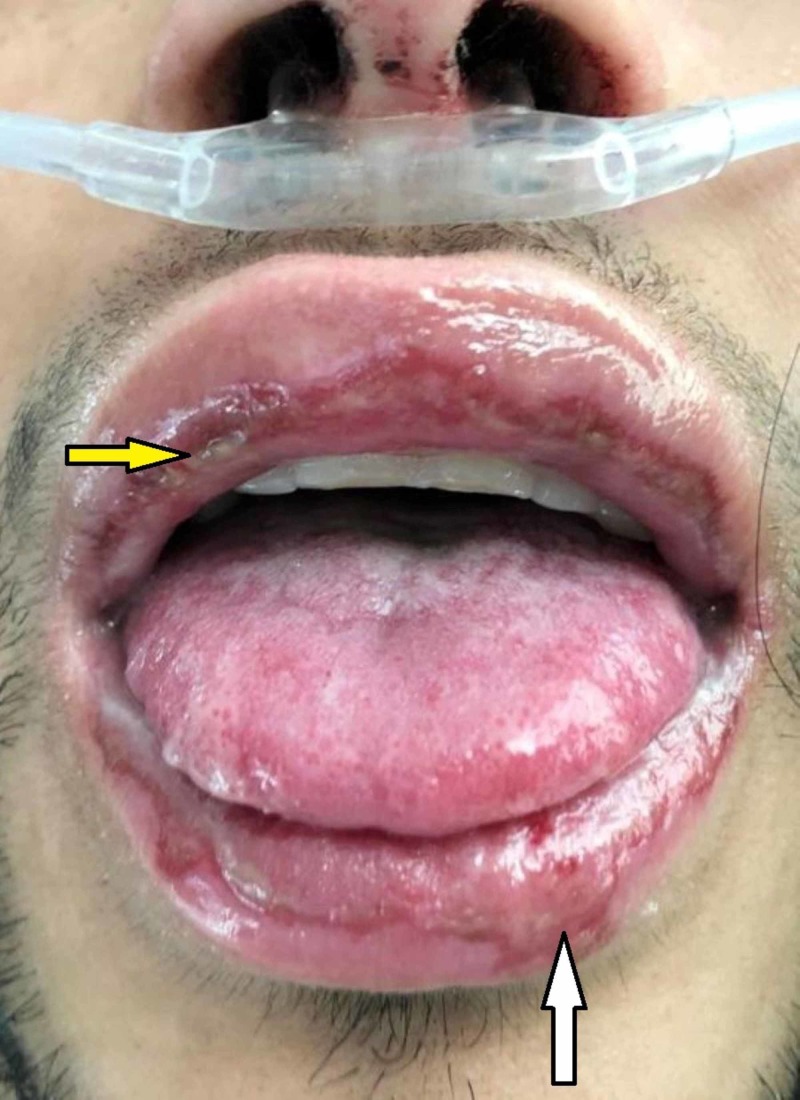
Initial facial examination Diffuse mucosal ulceration involving the dry mucosa of the upper (yellow arrow) and lower lips. There is also mild edema and hemorrhaging of the ulcerating edges (white arrow). Crusting along with dried blood was present on the nares and internally on the nasal septum.

**Figure 2:**
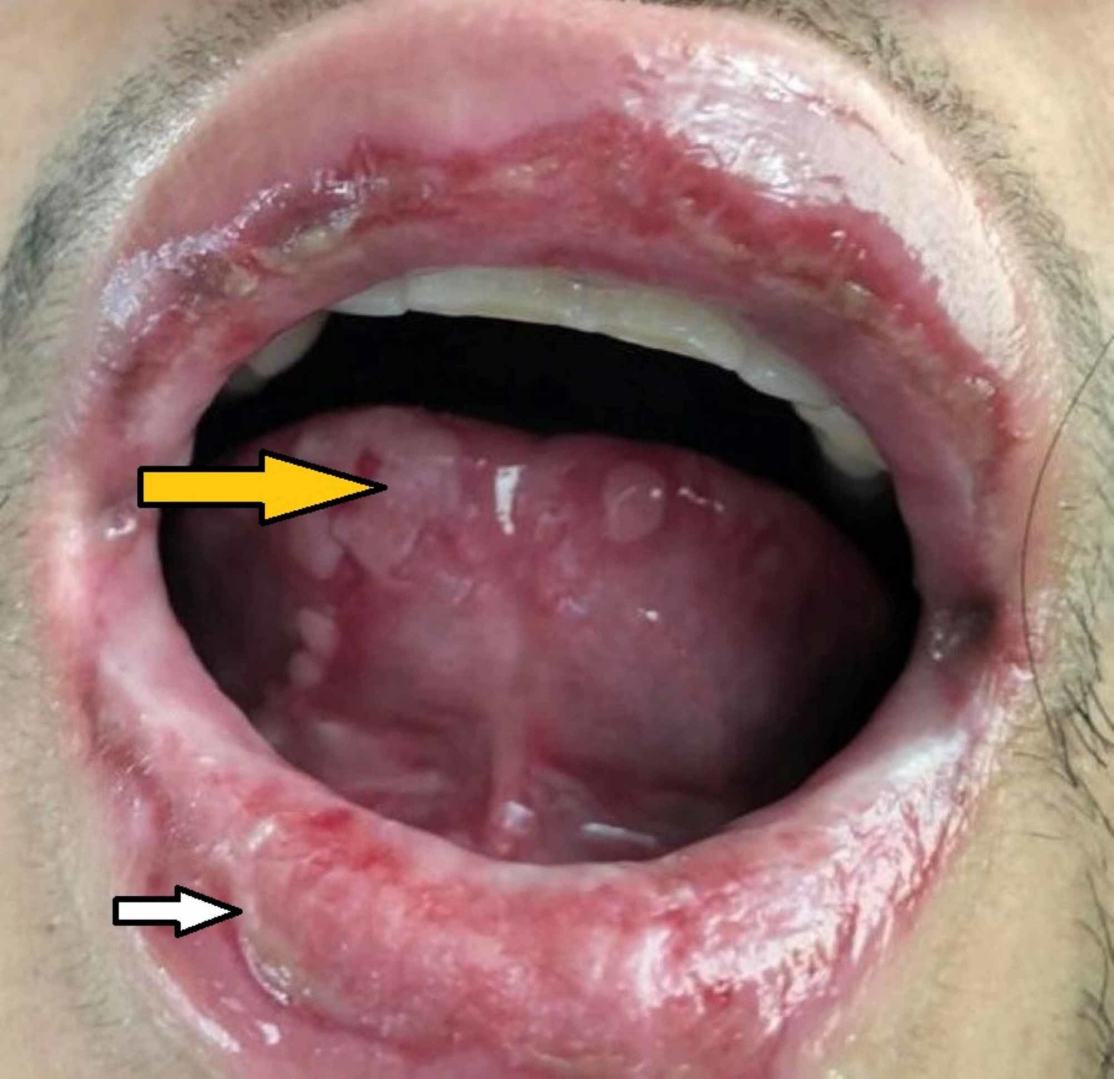
Buccal mucosa and anterior tongue Further diffuse mucosal ulceration of the upper and lower lip (white arrow). Note the diffuse bullous ulcerations of the ventral surface of the tongue (yellow arrow). This type of ulceration was present throughout the oral cavity, including the buccal mucosal surface and hard and soft palate, as well as the posterior oropharynx.

**Figure 3:**
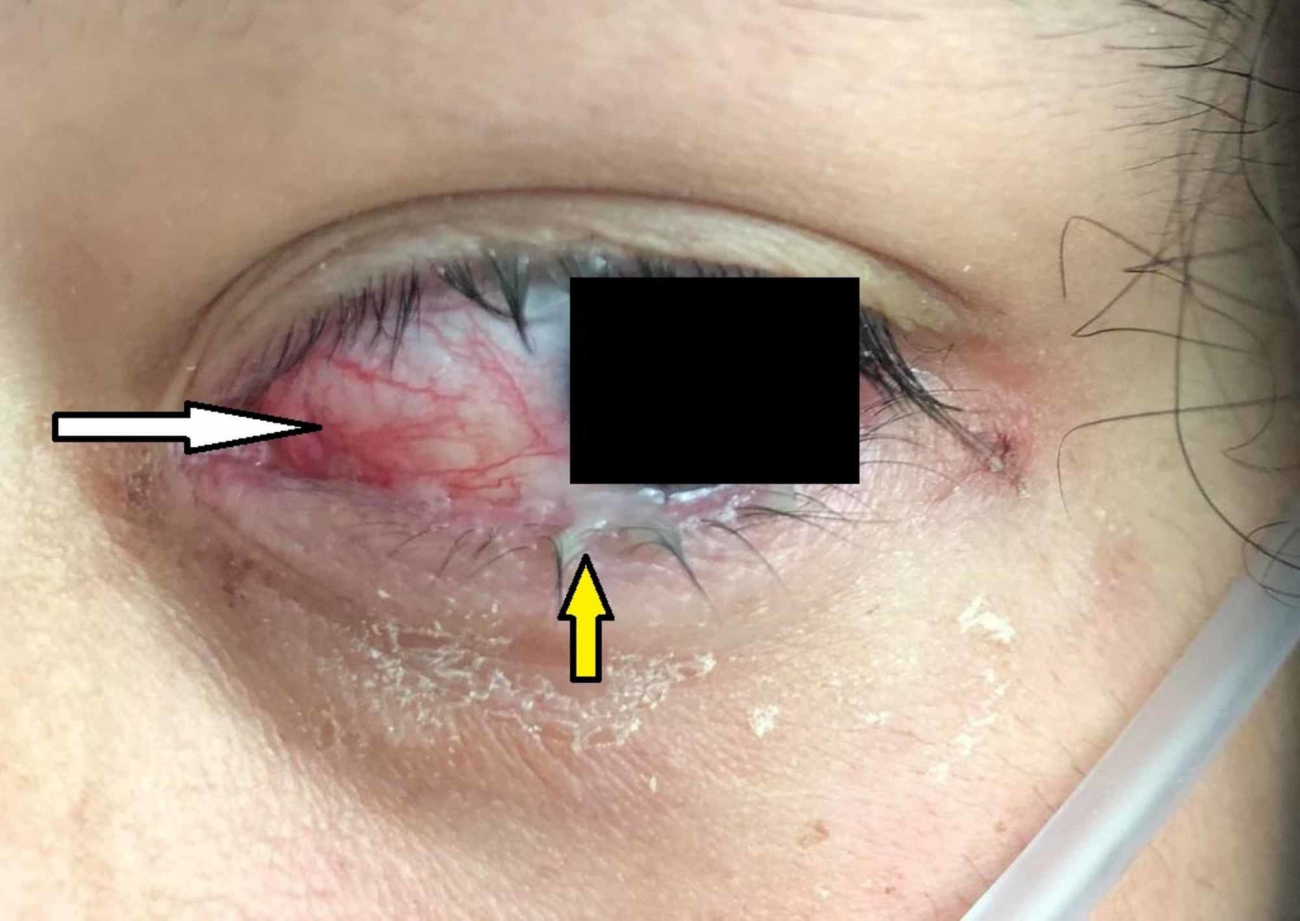
Ocular examination Initial ocular examination revealed bilateral purulent conjunctivitis. Note the pink-tinged conjunctiva (white arrow) with purulent discharge crusting around the lashes and lower eyelid (yellow arrow).

**Figure 4:**
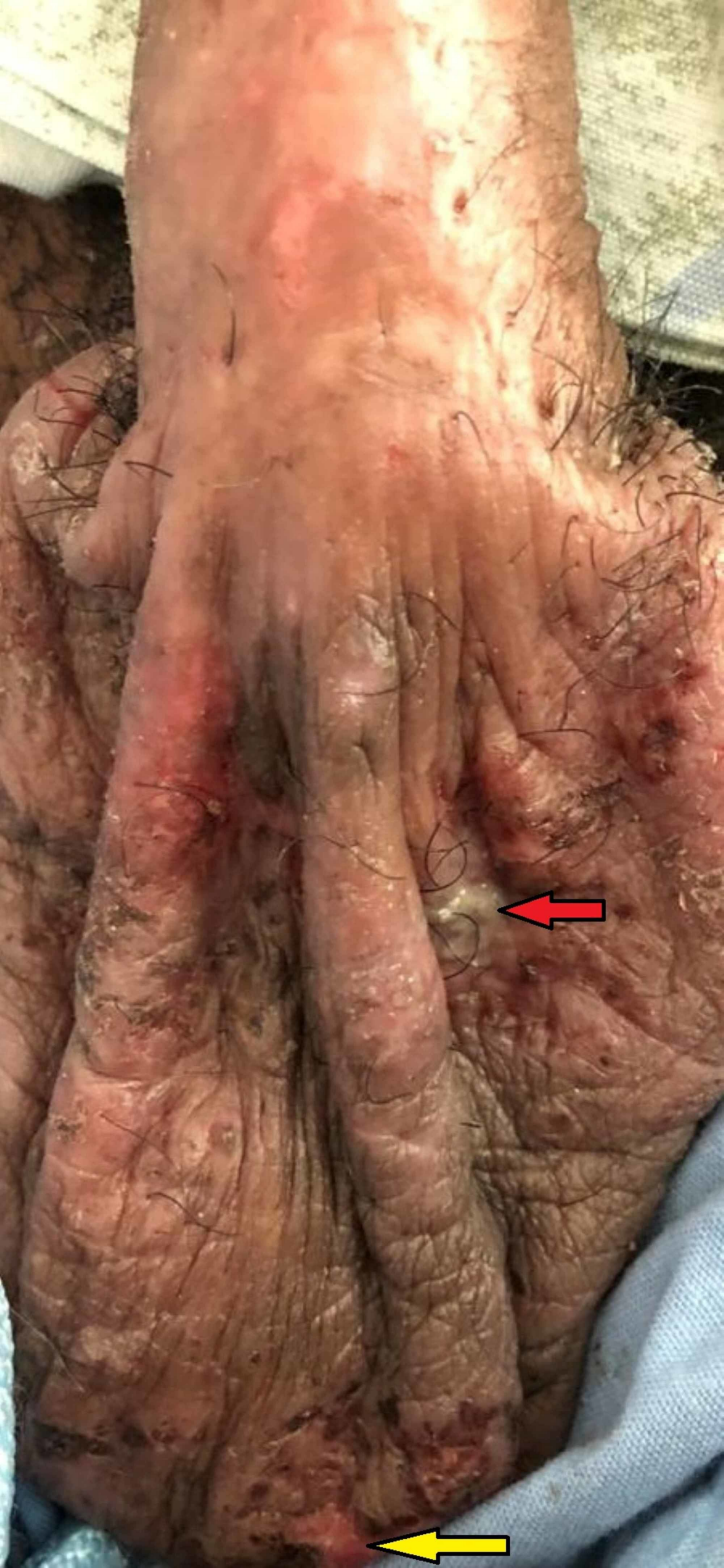
Genital lesions The patient suffered from painful, flat, and erythematous genital ulcerations. These either had notable purulence (red arrow) or crusted blood (yellow arrow) around their distinct borders.

Physical examination showed a patient in moderate distress with vital signs significant for tachycardia and oxygen saturation in the high 80s requiring high-flow oxygen through a non-rebreather mask. His pulmonary examination was significant for coarse breath sounds bilaterally and increased work of breathing. Oral examination revealed diffuse mucositis of the lips with significant ulceration of the tongue (hard and soft palate as seen in Figures 1 and 2, respectively). Left otoscopic examination revealed a 10% central perforation of the tympanic membrane with otorrhea. Right otoscopic examination revealed an intact tympanic membrane with purulent effusion suggestive of right otitis media. There was also bilateral conjunctivitis with purulent cloudy discharge, as seen in Figure 3. Nasal examination revealed crusting within the nares bilaterally without an acute source of epistaxis. Genitourinary examination revealed diffuse erythematous ulcerations of the scrotum, penile shaft, and glans, with occasional purulent discharge, as seen in Figure 4.

**Figure 5:**
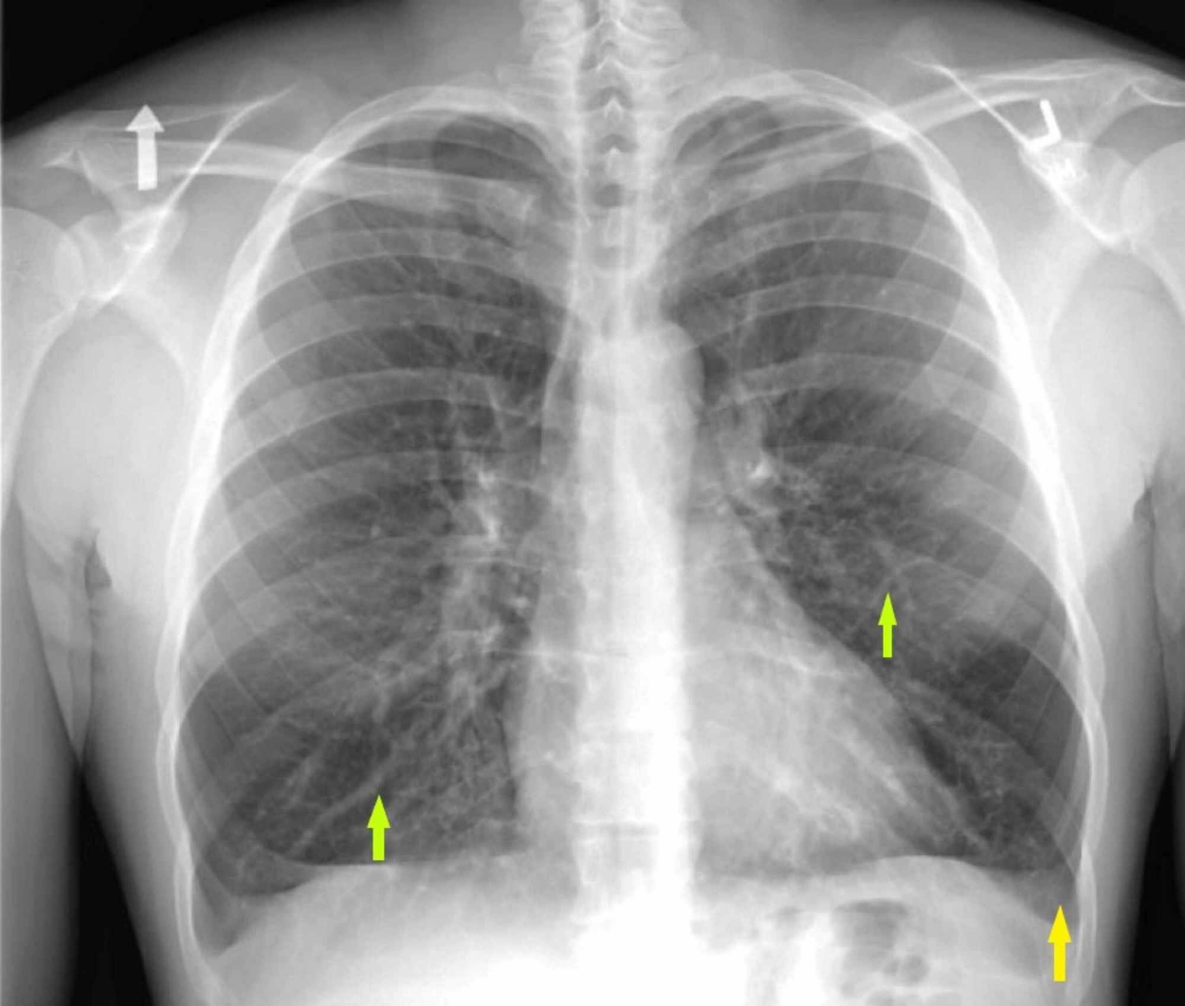
One-view chest radiograph Note the left-sided pleural effusion (yellow arrow) and bilateral diffuse interstitial infiltrates (green arrows).

**Figure 6:**
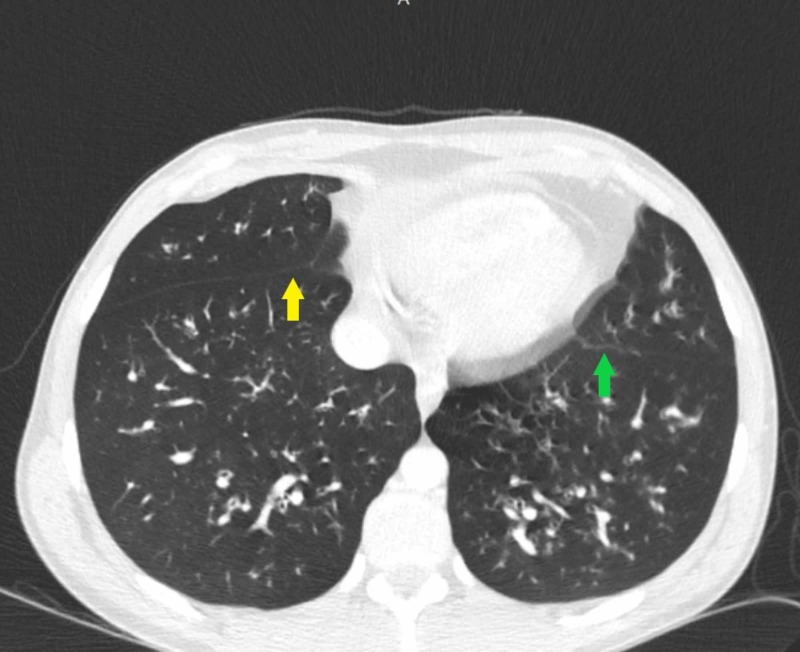
Contrasted CT scan of the lungs in an axial lung window Note the diffuse anterior and posterior ethmoid sinusitis (green arrow). Also note the accompanying sphenoid sinus thickening (yellow arrow).

**Figure 7:**
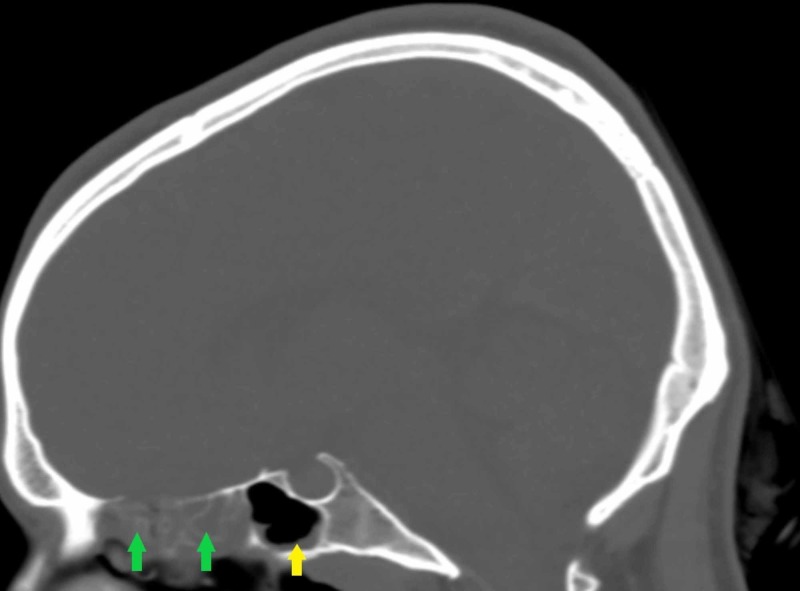
Non-contrasted CT scan of the head (sagittal plane) Note the diffuse anterior and posterior ethmoid sinusitis (green arrows). Also note the accompanying sphenoid sinus thickening (yellow arrow).

**Figure 8:**
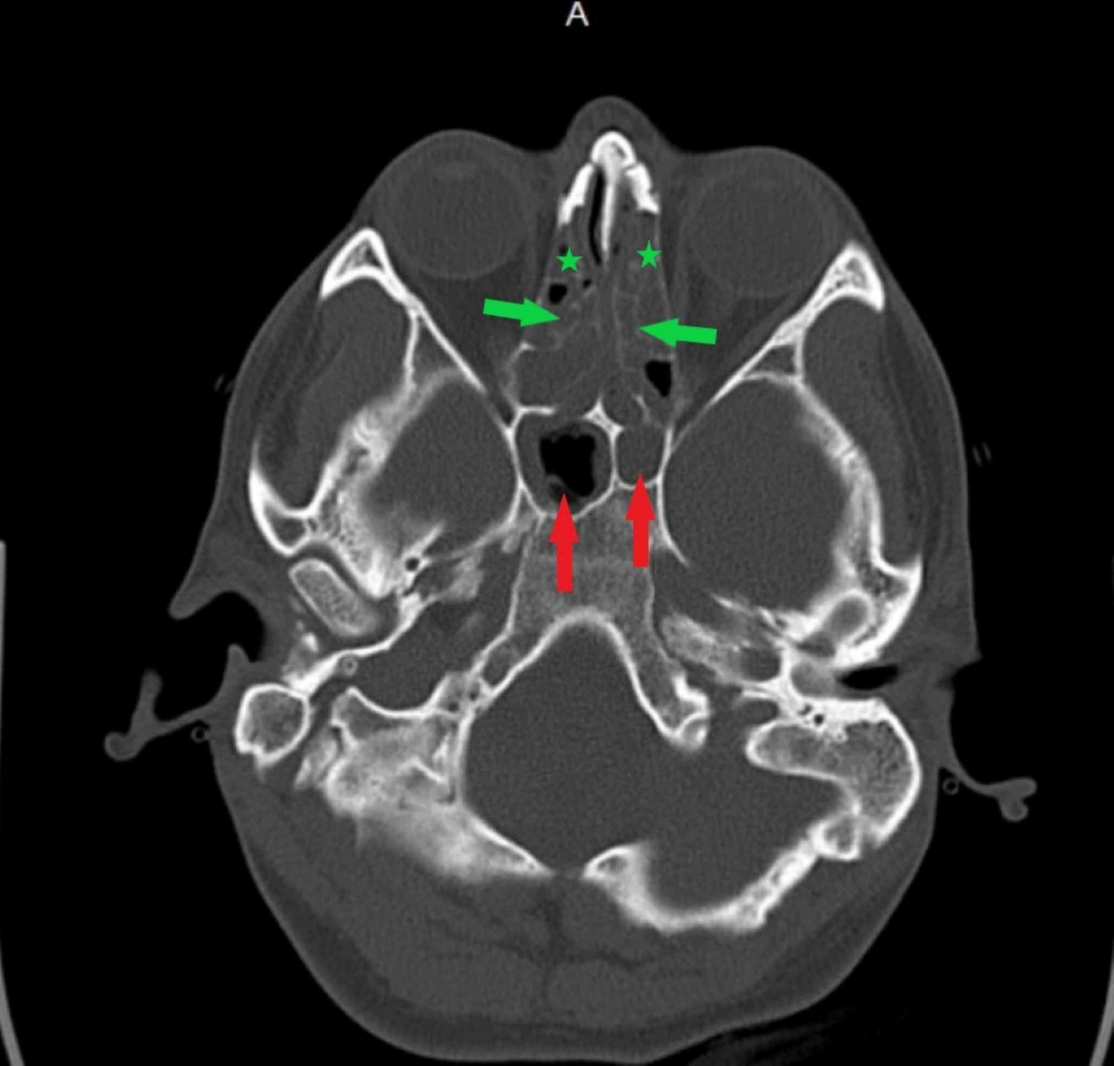
Non-contrasted CT scan of the head (axial plane) Note the diffuse anterior (green stars) and posterior (green arrows) ethmoid sinus disease. Also note the complete opacification of the left sphenoid sinus and moderate opacification of the right sphenoid sinus (red arrows).

**Figure 9:**
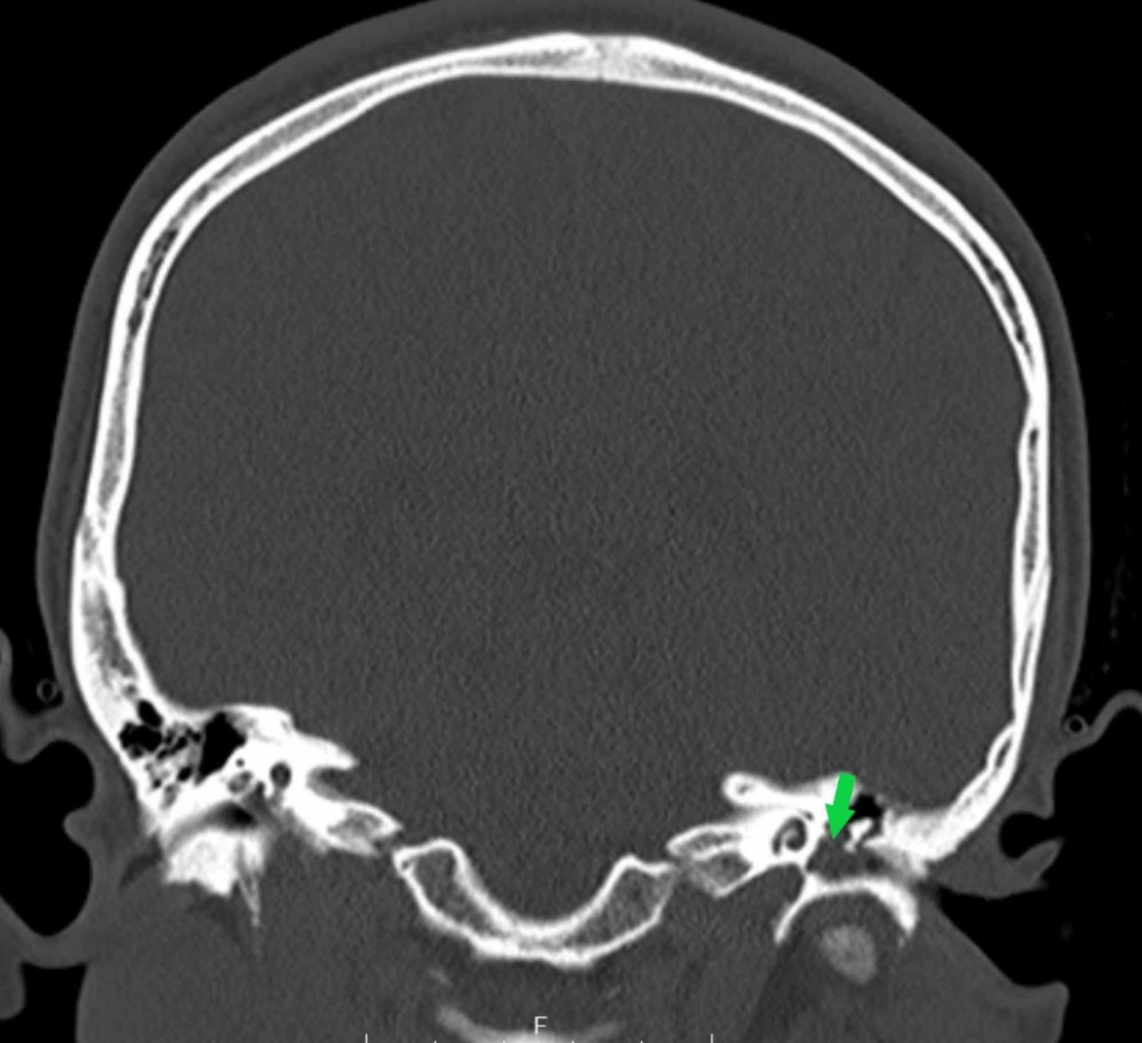
Non-contrasted CT scan of the head (coronal view) The green arrow points to the left middle ear space, which is almost completely opacified.

Initial lab results revealed an elevated CRP and leukocytosis with neutrophil predominance but were otherwise unremarkable. Initial chest X-ray showed bilateral pleural effusions and interstitial infiltrates, as seen in Figure 5. Contrasted computed tomography (CT) of the chest showed scattered ground-glass opacities most prominent in the lower lungs, as seen in Figure 6. Head CT showed moderate opacification of maxillary sinuses and complete opacification of the bilateral anterior and posterior ethmoid sinuses on both the sagittal and axial views, as seen in Figures 7 and 8, respectively. There was also opacification of the left middle ear space, with minimal mastoid involvement, as noted in Figure 9. The patient was then admitted to the intensive care unit (ICU) for acute hypoxic respiratory failure due to atypical pneumonia, and a full infectious and inflammatory workout was initiated. The patient was started on doxycycline, ceftriaxone, and fluconazole. Further testing was negative for human immunodeficiency virus (HIV), influenza, hepatitis B and C, syphilis, scleroderma, herpes simplex virus, Chlamydia, and Aspergillus. Both blood and sputum cultures were found to be negative; however, the patient was found to have elevated IgE and Mycoplasma IgM. It was then suspected at this time that the patient likely had MIRM.

The patient in total experienced 12 days of symptoms, with improvement in over three days of supportive care including pain control, fluid resuscitation, and local wound care, along with intravenous azithromycin and ceftriaxone. The patient had no lasting oral, ocular, genital, head, or neck sequelae.

## Discussion

In this report, we note the first published case of MIRM with both nasal and otologic symptomology. This reinforces the need for prompt otolaryngologic evaluation in patients suffering from epistaxis as well as otitis media with tympanic membrane rupture. The patient was treated with supportive management, the mainstay of which includes appropriate pain management, intravenous hydration, and mucosal care [3,5-7]. Two of the largest reviews of the literature indicate that 80% of MIRM patients were treated with antibiotics, 31-35% were treated with corticosteroids, 8-9% were treated with intravenous IGs (IVIG), and one patient was treated with plasmapheresis [3-6,10]. A recent case series of three patients with MIRM treated with cyclosporine A reported a statistically significant decrease in the overall length of hospital stay compared with supportive treatment in the literature [10]. Overall, patients suffering from MIRM tend to have longer hospital stays than patients suffering from EM alone and can require stays in the ICU or burn centers [3,5]. Our patient was admitted to the ICU but did not require intubation or burn center admission. Meyer Sauteur et al noted a statistically significant increase in hospital stay length (9.5 days) in patients suffering from MIRM compared with non-mycoplasma EM patients (5.1 days) with an odds ratio (OR) of 9. They also reported increased oxygen requirements in patients with MIRM versus CAP alone (OR=17.6) [6].

Although the majority of MIRM patients are generally known to have a full recovery (81%), a variety of complications have been noted in the literature. Orbital complications are noted in approximately 9% of patients and include corneal ulceration, blindness, conjunctival shrinkage, ocular synechiae, dry eyes, and lash loss. Post-inflammatory pigmentation changes are noted in about 5.6% of cases [5,6]. Oral and genital synechiae make up about 1% of complications each. Other rare reported complications include phimosis, vulvar adhesions, hematemesis, epiglottitis, B-cell lymphopenia, and death [3-5,8]. Only three cases of mortality due to MIRM were reported in the literature, but all notably took place prior to the 1940s. Genetic susceptibility has also been theorized to play a role due to the reported 8% recurrence rate and distribution in families as reported in the literature [5,11]. The patient in this report did not have any lasting complications or sequelae.

## Conclusions

In this report, we present the case of a 24-year-old male diagnosed with MIRM with ethmoid/maxillary sinusitis as well as unilateral tympanic membrane perforation secondary to bilateral otitis media. The authors hope that this report will lead to earlier identification of MIRM and its otolaryngologic manifestations while adding useful clinical information to a growing body of literature on the subject.
